# Near‐Infrared Spectroscopy (NIRS) Could Predict Graft Quality in Adult Liver Donors

**DOI:** 10.1155/joot/1261023

**Published:** 2026-06-19

**Authors:** Julen Ramón-Rodríguez, Diego López-Guerra, Noelia De Armas-Conde, Isabel Jaén-Torrejimeno, Adela Rojas-Holguín, María Ángeles Santiago-Triviño, Gerardo Blanco-Fernández, Demetrio Pérez-Civantos

**Affiliations:** ^1^ Department of HBP and Liver Transplant Surgery, University Hospital Complex Badajoz, University of Extremadura, Badajoz, Spain, unex.es; ^2^ Department of Health Sciences and Medicine, University of Extremadura, Badajoz, Spain, unex.es; ^3^ Department of Intensive Care Unit, University Hospital Complex Badajoz, University of Extremadura, Badajoz, Spain, unex.es

**Keywords:** liver transplant, near-infrared spectroscopy, steatosis

## Abstract

**Background:**

The use of livers from donors with expanded criteria, where monitoring the degree of hepatic steatosis (HS) is crucial, has increased in recent years to address the shortage of available grafts. The aim of this study is to evaluate the utility of monitoring regional hepatic oxygen saturation (rSO2) using the INVOS somatic oximeter in the assessment of liver donors.

**Methods:**

An observational, longitudinal, and prospective study was conducted of adult patients undergoing liver transplantation at our center between 08/01/2020 and 06/01/2022. We measured rSO2 by placing the sensor on the donor skin and on the liver surface.

**Results:**

The rSO2 measurements on the donor skin were obtained in 27 patients (93.1%), while the measurements on the donor liver were taken in 18 patients (62.1%). We found a statistically significant relationship between the rSO2 values measured on the donor liver and the degree of steatosis (*p* = 0.001). rSO2 values > 57% measured on the donor liver were associated with HS < 30% (S: 92%; E: 75%). Donors with lower preoperative GOT values had statistically significantly higher rSO2 values measured on the skin (*p* = 0.046).

**Conclusions:**

Hepatic rSO2 measurements obtained with the NIRS device in liver donors were associated with relevant donor graft characteristics, particularly the degree of HS.

## 1. Introduction

Liver transplantation (LT) is the treatment of choice for both acute and chronic end‐stage liver disease and also for certain malignancies. Around 25,000 liver transplants are performed each year worldwide. In recent decades, survival rates for liver transplant recipients have improved, with the 1‐year survival rate currently at 90% [1]. This increase is due to multiple factors, including better selection of donors and recipients, standardization of surgical technique, better intraoperative patient control, advances in immunosuppression and organ preservation solutions, and better management of postoperative complications [2].

As a result of these improved outcomes, the indications for LT have been expanded. This, coupled with a decline in graft availability due to reduced mortality from traffic accidents, has led to a significant gap between supply and demand [3, 4]. To address the liver shortage, the criteria for the use of livers have been expanded to include organs from donors after circulatory death (DCD) and also elderly donors. In donors of this kind, control of hepatic steatosis (HS) is essential in order to obtain optimal results [5–7].

In several studies, the presence of HS > 30% in donor livers has been associated with worse outcomes, with reduced survival of both the recipient and the graft and increased postoperative complications [8, 9].

In the process of the evaluation of the donor liver, significant discrepancies have been observed between the subjective opinion of the surgeon regarding the degree of HS and the results of the pathology study of the liver biopsy. Thus, livers that may appear acceptable to the surgeon at macroscopic level may present high degrees of steatosis in the microscopic analysis [10].

In recent years, the use of near‐infrared spectroscopy (NIRS) systems, such as the INVOS device (Covidien, Minneapolis, MN, USA), has become more widespread. This device measures the regional oxygen saturation (rSO2) of a tissue using infrared light thanks to the different degree of absorption of light by oxygenated and deoxygenated hemoglobin. Initially, this device was used to measure cerebral rSO2 during cardiovascular surgery, improving postoperative outcomes; more recently, it has been used to monitor kidney grafts during the immediate postoperative phase [11–13]. Regarding LT, recent publications have demonstrated the relationship between low hepatic rSO2 values in recipients in the immediate postoperative period and the appearance of early complications such as graft failure and vascular complications [14–16].

The aim of the present study is to determine the usefulness of monitoring hepatic rSO2 using the device in the assessment of liver donors.

## 2. Materials and Methods

### 2.1. Patient Selection

We conducted a prospective longitudinal observational study of adult patients undergoing LT at our center between 08/01/2020 and 06/01/2022.

- The inclusion criteria were as follows: liver grafts from valid adult donors transplanted at our center.
- The exclusion criteria were as follows: livers from donors in whom none of the measurements under study could be performed and grafts that were rejected during donation and were not implanted.

### 2.2. Variables

The following donor variables were studied: age, sex, body mass index (BMI), diabetes mellitus, cause of death, DCD, donor origin, intensive care unit (ICU) stay, last value of GOT, GPT, and plasma sodium, Donor Risk Index (DRI), and hepatic rSO2 measured on the skin and liver.

The presurgical data of the recipients studied were as follows: age, sex, BMI, indication for LT, Model for End‐Stage Liver Disease (MELD), and MELD‐Na.

The perioperative variables studied in the recipients were as follows: ischemia time, need for intraoperative norepinephrine, ICU stay, SOFA and APACHE II scores, International Normalized Ratio (INR), maximum total bilirubin on the first postoperative day, maximum GOT and GPT in the postoperative period, and degree of HS and ischemia‐reperfusion damage measured in the time‐zero biopsy.

HS was classified based on the number of hepatocytes affected by fat vacuoles in the pathology study and was defined as absent, mild (10%–30%), moderate (30%–60%), and severe (> 60%). Donor livers in our sample with a level of steatosis of up to 30% in the time‐zero biopsy were assigned to the absent–mild steatosis group, and those with values of 30% or higher were assigned to the moderate–severe group. The cutoff point of 30% was used because above this value, the results of LT are reported to worsen notably [8, 9].

The DRI was determined using the model described by Feng et al. based on the following donor values: age, height, cause of death, ethnicity, cardiac death, split graft, origin of the liver, and cold ischemia time [17]. Donor data were obtained from the National Transplant Organization database and recipient data from our center’s computer system.

### 2.3. Donor Assessment

The assessment and decision to accept the donor livers included in the study was carried out by members of our center’s liver transplant team. Liver grafts harvested at external centers by other teams were excluded from the study because measurements were not performed. In case of doubt about the viability of the graft, an intraoperative liver biopsy was performed before confirming its validity. rSO2 measurements were not incorporated into the decision‐making process for accepting or rejecting the liver.

### 2.4. NIRS Measurement Technique

Hepatic rSO2 measurements are performed with the INVOS 5100C device (Covidien, Minneapolis, MN, USA). This NIRS system has a penetration of approximately 3 cm.

A measurement is performed on the donor before starting the extraction surgery, placing the sensor on the skin at the level of the right hypochondrium, just above the liver. Later, during surgery, with the donor liver exposed, a second sterile measurement is performed using a plastic sheath covering the device cable. To do this, the device sensor is placed on the surface of the liver, in segment 4b at the level of the portal bifurcation. To allow this second measurement, we spread a layer of sterile conductive gel on the liver surface under study and we reduce light contamination in the operating room. This procedure is performed by the surgeons of the team in charge of the donation who are all also members of our hospital’s transplant team.

Subsequently, all valid livers are transplanted by our hospital’s surgical team. At the end of the LT, a biopsy of the liver graft is taken, which is sent to the Pathology Service for analysis. The results of the pathology study indicate the degree of steatosis and the degree of ischemia‐reperfusion damage of the liver graft.

### 2.5. Ethics Statement

The present study was approved by our hospital’s Research Ethics Committee. The requirement for informed consent was waived owing to its observational nature.

### 2.6. Statistical Analysis

The data from our study were analyzed using SPSS statistics software Version 21 (IBM, Armonk, New York). Means and standard deviations (SDs) were used to describe the quantitative variables with normal distributions, and medians and interquartile ranges (IQRs) were used to describe those with nonnormal distributions. For the study of qualitative variables, the frequency distributions and percentages (%) were used.

To assess the association between a binary categorical variable and quantitative variables, Student’s *t*‐test was used. To assess the association between two quantitative variables, we used Pearson’s correlation and the linear regression test. The statistical results were considered statistically significant when *p* < 0.05 was obtained.

To establish the cutoff point of rSO2 with the highest sensitivity and specificity for the appearance of HS, we used the receiver operating characteristic (ROC) curve and the Youden index.

Univariate binary logistic regression analyses were performed to identify donor‐related variables associated with HS. Variables with *p* < 0.20 in univariate analysis were considered for multivariable modeling.

## 3. Results

**FIGURE 1:**
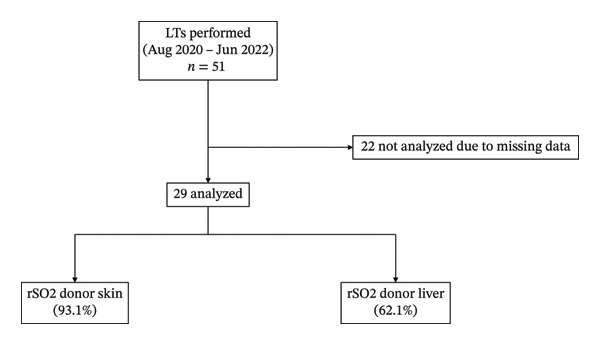
Flowchart of the study cohort.

Between August 2020 and June 2022, a total of 51 patients underwent liver transplant at our center. Twenty‐two of these patients were excluded from the study because in their case, none of the required measurements were performed, either because the liver was harvested at an external center or because a super‐rapid harvest was performed in donors in controlled type 3 asystole by our surgical team. Finally, a total of 29 recipients and their respective matched donors were included in the analysis. Not all of the data under study were available in all patients: rSO2 on donor skin was obtained in 27 patients (93.1%) and on the donor liver in 18 (62.1%) (Figure 1).

The characteristics of the donors are summarized in Table 1; the baseline characteristics of the recipients are summarized in Table 2, and the intra‐ and postoperative variables corresponding to the recipients are listed in Table 3.

**TABLE 1:** Baseline characteristics of the donors in the cohort.

Donors	Mean (SD)/median (IQR) or frequency (%)
Age (years)	67 (47–76)
Sex (male/female)	18/11 (62.1/37.9)
BMI (kg/m^2^)	25 (22.4–27.7)
Diabetes mellitus 2 (No/Yes)	20/9 (69/31)
Cause of death	
CVA	20 (69)
TBI	6 (20.7)
Others	3 (10.3)
Asystole (No/Yes)	19/10 (65.5/34.5)
Origin	
Local	15 (51.7)
Regional	7 (24.1)
External	7 (24.1)
Need for vasoactive drugs (No/Yes)	11/18 (37.9/62.1)
ICU stay (days)	4 (1–6)
GOT (IU/L)	29 (18–50)
GPT (IU/L)	23 (13–34)
Plasma sodium (mEq/L)	142 (138–149)
DRI	1.99 (0.38)
rSO2 donor skin (%)	78 (71–86)
rSO2 donor liver (%)	66.4 (12.1)

*Note:* CVA: cerebrovascular accident; IQR: interquartile range; rSO2: regional oxygen saturation.

Abbreviations: BMI, body mass index; DRI, Donor Risk Index; GOT, glutamic oxaloacetic transaminase; GPT, glutamic pyruvic transaminase; ICU, intensive care unit; SD, standard deviation; TBI, traumatic brain injury.

**TABLE 2:** Baseline characteristics of the recipients in the cohort.

Recipients	Mean (SD)/median (IQR) or frequency (%)
Age (years)	58.9 (8)
Sex (male/female)	23/6 (79.3/20.7)
BMI (kg/m^2^)	25.5 (23.5–27.7)
Liver disease etiology	
Hepatocellular cirrhosis	22 (75.9)
Cholestatic disease	3 (10.3)
Autoimmune hepatitis	2 (6.9)
Hepatorenal polycystosis	2 (6.9)
MELD	12 (9.5–16)
MELD‐Na	14.9 (4.8)

*Note:* IQR: interquartile range; MELD, Model for End‐Stage Liver Disease; MELD‐Na: Model for End‐Stage Liver Disease‐Sodium.

Abbreviations: BMI, body mass index; SD, standard deviation.

**TABLE 3:** Intra‐ and postoperative characteristics of the study cohort.

Intra and postoperative	Mean (SD)/median (IQR) or frequency (%)
Total ischemia time (minutes)	348.8 (59.9)
Cold ischemia time (minutes)	316.7 (57.6)
Warm ischemia time (minutes)	32.1 (4.7)
Intraoperative norepinephrine (No/Yes)	10/19 (34.5/65.5)
ICU stay (days)	3 (2.5–4)
SOFA	4.93 (1.9)
APACHE II	12 (9–13)
INR	1.72 (0.4)
Bilirubin (mg/dL)	2.6 (1.4–4)
GOT (IU/L)	594 (414–1138)
GPT (IU/L)	462 (269–1023)
Degree of steatosis (%)	5 (5–20)
Ischemia‐reperfusion damage:	
Mild	15 (51.7)
Moderate	13 (44.8)
Severe	1 (3.4)

*Note:* APACHE II: Acute Physiology and Chronic Health Disease Classification System II; IQR: interquartile range.

Abbreviations: GOT, glutamic oxaloacetic transaminase; GPT, glutamic pyruvic transaminase; ICU, intensive care unit; INR, International Normalized Ratio; SD, standard deviation; SOFA, Sequential Organ Failure Assessment.

The study included donor livers procured nationwide, encompassing our center, regional hospitals, and other institutions across the country, as reflected in Table 1.

**FIGURE 2:**
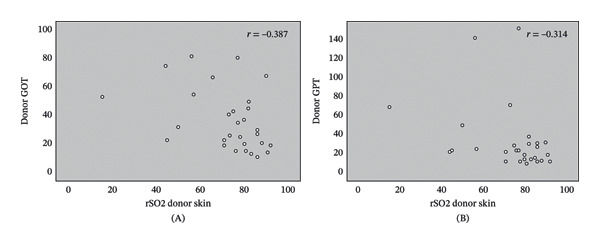
(A) Scatter plot and linear regression of hepatic rSO2 on donor skin and the last donor GOT value. (B) Scatter plot and linear regression of hepatic rSO2 on donor skin and the last donor GPT value.

A statistically significant relationship was obtained between donors’ last GOT values and the hepatic rSO2 values measured on their skin; those with lower preoperative GOT rates had higher rSO2 values (*p* = 0.046) (Figure 2A).

This same trend toward higher rSO2 values on donor skin in patients with lower transaminase values was also observed in the case of GPT, although the difference was not significant (*p* = 0.110) (Figure 2B).

**FIGURE 3:**
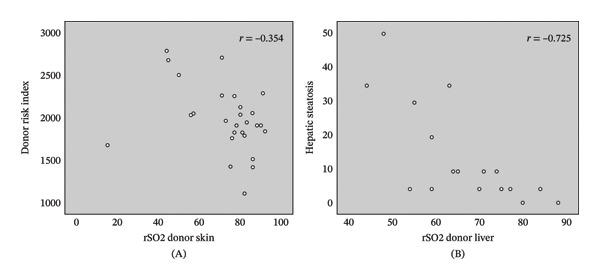
(A) Scatter plot and linear regression of hepatic rSO2 on donor skin and DRI. (B) Scatter plot and linear regression of hepatic rSO2 on donor liver and degree of HS at time 0 biopsy.

A trend was also observed toward higher rSO2 values on donor skin in patients with a lower DRI, although the results did not reach statistical significance (*p* = 0.07) (Figure 3A).

There was a statistically significant association between the degree of HS of the donor liver measured at time‐zero biopsy and the rSO2 values on the donor liver (*p* = 0.001). Patients with lower rSO2 values had a higher degree of HS in the subsequent pathology study (Figure 3B).

**FIGURE 4:**
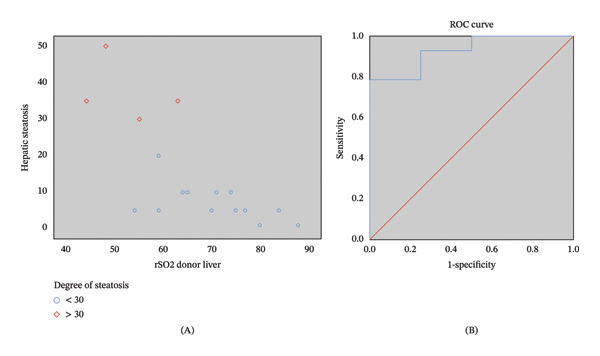
(A) Scatter plot of hepatic rSO2 on the donor liver and the degree of steatosis at time 0 biopsy, divided based on the degree of steatosis (< 30% or ≥ 30%). (B) ROC curve analyzing rSO2 on liver donors with a degree of hepatic steatosis < 30% in the time 0 biopsy.

Patients in the moderate–severe HS group (≥ 30%) had poorer rSO2 results than those in the absent–mild group (< 30%) (*p* = 0.005) (Figure 4A).

We performed a ROC curve and used the Youden index to check the rSO2 value above which the livers were more likely to belong to the absent–mild steatosis group. We found that livers with rSO2 values > 57% on the donor liver were associated with a degree of HS below 30% with a sensitivity of 92% and specificity of 75%. The area under the curve for this test was 0.929 (CI 95%: 0.803–1) (Figure 4B).

In univariate analysis, donor BMI, skin rSO_2_, and hepatic surface rSO_2_ were associated with the degree of HS (*p* < 0.20) (Table 4). However, multivariable logistic regression including all three variables resulted in model instability due to complete separation and was therefore not performed.

**TABLE 4:** Univariate analysis of variables associated with the degree of hepatic steatosis.

Variable	*p* value	*β* coefficient
Age	0.333	0.138
BMI	0.024	0.780
Diabetes	0.830	1.189
Asystole	0.335	−5.142
GOT	0.471	−0.045
GPT	0.505	−0.040
rSO2 donor skin	0.156	0.206
rSO2 donor liver	0.001	−0.843

*Note:* rSO2: regional oxygen saturation.

Abbreviations: BMI, body mass index; GOT, glutamic oxaloacetic transaminase; GPT, glutamic pyruvic transaminase.

No statistically significant correlation was found between skin and hepatic surface rSO2 measurements (*r* = 0.30, *p* = 0.25).

## 4. Discussion

In recent decades, we have witnessed increases in the survival of both liver recipients and grafts, which has led to an expansion of the indications for LT. In turn, this, however, has resulted in a new challenge: a shortage of available organs. The response has been to expand the donor criteria, accepting livers that a few years ago would have been rejected—for instance, organs from elderly donors and DCDs [18]. Several studies have shown that to obtain satisfactory results with livers from these donors, controlling the cold ischemia time and the degree of HS are priority issues [7, 19].

The decision to accept or reject a liver graft has far‐reaching consequences; it is based on the clinical, analytical, and, above all, morphological criteria of the graft assessed intraoperatively.

Recently, the indications of infrared light systems to assess the rSO2 of an organ have also broadened substantially. Initially used at the brain level, their applications now extend to the kidney and liver [12, 14, 20, 21].

Our study is the first of its kind to assess the usefulness of NIRS systems for evaluating livers during the donation process, and it has demonstrated a relationship between the levels of GOT in the donor and the values of hepatic rSO2 obtained on the donor’s skin (*p* = 0.046). The higher the donor’s serum GOT levels, the lower the rSO2 of the liver; thus, NIRS may be a useful real‐time guidance tool for assessing transaminase levels. This relationship between transaminases and hepatic rSO2 may be attributable to ischemia and the damage thus caused to liver cells, with the resulting elevation of transaminases, with the ischemic process being detectable by the NIRS system.

The donors in our study with higher DRI values presented a trend toward worse values of hepatic rSO2 measured on the skin (*p* = 0.07). This association did not reach statistical significance, possibly due to the low number of patients in our study, but it indicates that NIRS may give an idea of the risk associated with a particular donor and of the predictive data provided by the DRI, an internationally validated scale.

The surgeon’s subjective evaluation of the liver graft for determining the degree of HS has been shown to be inaccurate since, in many cases, it does not correspond to the objective result provided by the liver biopsy. This means that the intraoperative biopsy is the gold standard for evaluating the degree of HS, but it is not always available [10]. In our study, we found a statistically significant relationship between the rSO2 values measured on the surface of the donor liver and the degree of HS in the time‐zero biopsy. We verified that the higher the degree of HS in the pathology study, the lower the rSO2 levels obtained in the measurement on the liver (*p* = 0.001). The rSO2 reflects the balance between oxygen delivery and consumption at the microcirculatory level and is therefore sensitive to structural and functional alterations of the hepatic microvasculature. In the context of HS, sinusoidal compression by enlarged, fat‐laden hepatocytes and lipid‐related microvascular dysfunction impair intrahepatic perfusion, leading to reduced rSO2. Consequently, the observed decrease in hepatic rSO2 in steatotic grafts likely represents the detection of underlying microcirculatory disturbances associated with pathological fat infiltration [8].

Patients in the moderate/severe steatosis group had worse rSO2 results than those in the absent/mild group (*p* = 0.005). The cutoff value for rSO2 was 57%, meaning that donor livers with rSO2 values on their surface of > 57% are more likely to have absent–mild steatosis (sensitivity: 92%; specificity: 75%). The proposed rSO2 cutoff of 57% should be interpreted with caution, as it is derived from a small sample size and must be considered exploratory and hypothesis‐generating rather than clinically definitive.

In univariate linear regression analysis, hepatic surface rSO2 was inversely associated with the degree of steatosis at time‐zero biopsy (*β* = −0.84% per 1% increase in rSO2, *p* = 0.001). Accordingly, each 10% increase in hepatic surface rSO2 was associated with an approximately 8% lower degree of histological steatosis. The strong inverse relationship between hepatic surface rSO2 and histological steatosis supports the hypothesis that NIRS‐derived measurements reflect microcirculatory alterations associated with fatty infiltration of the graft.

On the other hand, the poor correlation between skin and hepatic surface rSO2 measurements may be explained because skin measurements are influenced by systemic hemodynamic conditions and subcutaneous tissue characteristics, whereas direct hepatic surface measurements more specifically reflect intrahepatic microcirculatory status.

Thus, measuring rSO2 on the donor liver has potential to serve as a noninvasive way of obtaining instant, quantitative, and dynamic information on the degree of steatosis of the future graft. It is well known that the degree of HS is a key factor in ensuring the correct functioning of liver grafts, especially those from elderly or asystole donors, which are increasingly used today [5–7]. Furthermore, in the context of the global obesity epidemic, it will be increasingly common to find donors in routine clinical practice who are overweight and, consequently, have a higher degree of HS [22].

The findings of this study indicate that NIRS could serve as an additional tool for evaluating graft quality, thus shortening the decision‐making time. Among the advantages of the NIRS device are the fact that it is a small, easy‐to‐transport medical device that performs noninvasive and continuous measurements and is available in many third‐level hospitals.

The main limitation of our study is the small sample size because some of the liver grafts were harvested at external hospitals and no measurements were carried out. Owing to the limited sample size and the resulting instability of multivariable models, a multivariable analysis could not be reliably performed. Therefore, the present findings should be interpreted as preliminary and require validation in larger, independent cohorts. Another limitation is that the study was carried out on implanted livers, and grafts rejected during donation were not assessed. This study was specifically designed to characterize viable livers, rather than as a tool for decision‐making regarding organ suitability. Likewise, no pathological differentiation was made between microvesicular and macrovesicular steatosis. However, in spite of these shortcomings, our results are promising and lay the foundations for future research with measurements on the liver surface using infrared spectroscopy systems. Additionally, due to the 3‐cm penetration depth of the NIRS device, its use may not be recommended in patients with obesity.

We stress that our results are preliminary and must be confirmed by studies with larger patient populations.

## 5. Conclusions

Hepatic rSO2 measurements obtained with the NIRS device in liver donors were associated with relevant donor and graft characteristics, particularly the degree of HS. These findings suggest that NIRS‐derived hepatic surface rSO2 reflects underlying microcirculatory alterations related to fatty infiltration and may provide complementary, real‐time information during donor evaluation.

**Nomenclature**
BMI: Body mass index
DCD: Donors after circulatory death
GOT: Glutamic oxaloacetic transaminase
GPT: Glutamic pyruvic transaminase
HS: Hepatic steatosis
ICU: Intensive care unit
INR: International Normalized Ratio
IQR: Interquartile range
LT: Liver transplantation
MELD: Model for End‐Stage Liver Disease
NIRS: Near‐infrared spectroscopy
ROC: Receiver operating characteristic
rSO2: Regional oxygen saturation
SD: Standard deviation

## Author Contributions

Julen Ramón‐Rodríguez: conceptualization, methodology, investigation, formal analysis, and writing–original draft; Diego López‐Guerra: conceptualization, methodology, investigation, formal analysis, and writing–original draft; Noelia De Armas‐Conde: validation, writing–review and editing, and visualization; Isabel Jaén‐Torrejimeno: validation, writing–review and editing, and visualization; Adela Rojas‐Holguín: validation, writing–review and editing, and visualization; María Ángeles Santiago‐Triviño: validation and investigation; Gerardo Blanco‐Fernández: investigation, writing–review and editing, visualization, and supervision; and Demetrio Pérez‐Civantos: investigation, writing–review and editing, visualization, and supervision.

## Funding

This research did not receive any specific grant from funding agencies in the public, commercial, or not‐for‐profit sectors.

## Disclosure

This manuscript has been presented in The Liver Meeting: 2025 in the categories: donor and allocation issues, living donor and split liver transplantation, and hepatobiliary surgery.

## Ethics Statement

This study was approved by the Ethical Committee of our institution.

## Conflicts of Interest

The authors declare no conflicts of interest.

## Data Availability

The relevant data are real and available from the corresponding author upon request.
